# 1-Benzyl-3-methyl­imidazolium bromide

**DOI:** 10.1107/S2414314620007683

**Published:** 2020-06-09

**Authors:** Tim Peppel, Christoph Wulf, Anke Spannenberg

**Affiliations:** a Leibniz-Institut für Katalyse e. V., Albert-Einstein-Str. 29a, 18059 Rostock, Germany; University Koblenz-Landau, Germany

**Keywords:** crystal structure, ionic liquid, hydrogen bond, imidazolium

## Abstract

The title compound, (BenzMIm)Br (BenzMIm=1-benzyl-3-methyl­imidazo­l­ium), C_11_H_13_N_2_^+^·Br^−^, was obtained as single crystals directly from a pure and liquid sample of the compound over several weeks. The mol­ecular structure of (BenzMIm)Br consists of separated bromide anions and 1-benzyl-3-methyl­imidazolium cations connected *via* short C—H⋯Br contacts. The compound exhibits a relatively low melting point (m.p. = 72°C) and is a supercooled, highly viscous transparent liquid at ambient conditions. The title compound crystallizes with two unique ion pairs in the asymmetric unit of the orthorhombic unit cell.

## Abstract 2

The mol­ecular structure of the title compound, (BenzMIm)Br (BenzMIm=1-benzyl-3-methyl­imidazolium), consists of separated bromide anions and 1-benzyl-3-methyl­imidazolium cations connected *via* short C—H⋯Br contacts.

## Graphical abstract

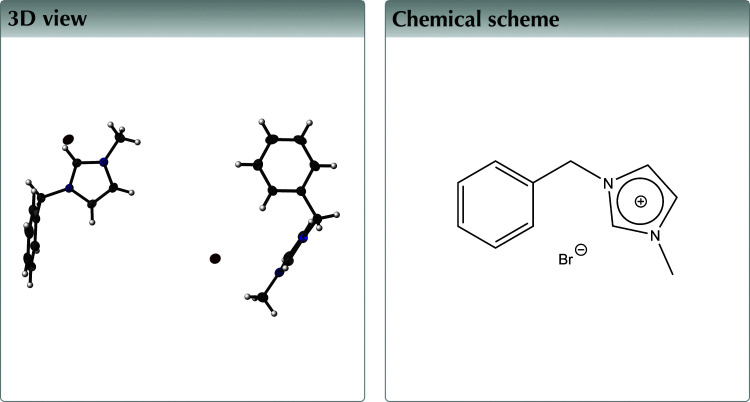

## Structure description

**Figure 1:**
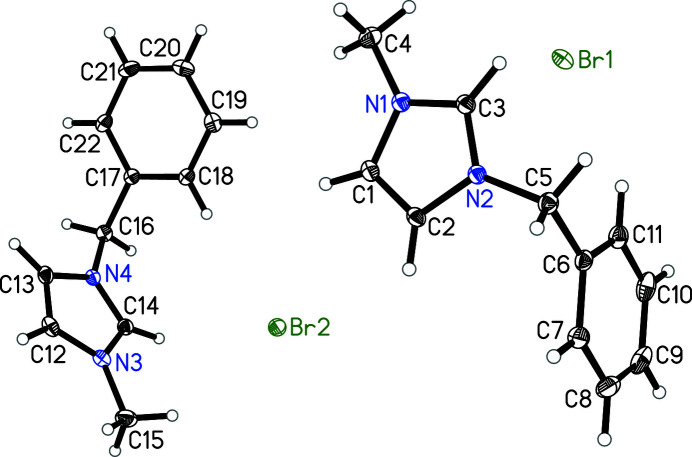
Mol­ecular structure of the title compound. Displacement ellipsoids correspond to 30% probability.

For the last 20 years, ionic liquids as salts with low melting points have attracted great inter­est because of their unique properties and applications. These properties include, for instance, large liquid ranges, broad electrochemical windows as well as low vapor pressures (Hallett & Welton, 2011 ▸; Welton, 1999 ▸). The title compound, which is a useful starting material in our ongoing efforts to investigate metal-containing ionic liquids (Peppel *et al.*, 2010 ▸; Peppel *et al.*, 2017 ▸; Peppel *et al.*, 2019 ▸) was obtained as single crystals over a period of several weeks directly from its pure, highly viscous and supercooled liquid. 1-Benzyl-3-methyl­imidazolium bromide expands the range of known single-crystal X-ray structures of ionic liquids of the general formula (BenzMIm)*X* (*X* = Cl, PF_6_ (Ji *et al.*, 2010 ▸; Hillesheim & Scipione, 2014 ▸) with a third example (*X* = Br). It can be seen from Fig. 1 ▸ that the (BenzMIm)Br is characterized by discrete 1-benzyl-3-methyl­imidazolium cations and bromide anions. The shortest C—H⋯Br contacts equal 2.740 Å (sum of van der Waals radii for H and Br: 3.0 Å). All bond lengths and angles within the cation are in expected ranges (Leclercq *et al.*, 2009 ▸). The two symmetry-independent mol­ecular units mainly differ by the angle between the phenyl and the imidazolium ring which is 84.02 (7)° in one of the cations and to 80.47 (7)° in the other. The title compound crystallizes with two unique ion pairs in the asymmetric unit of the orthorhombic unit cell.

## Synthesis and crystallization

The title compound, (BenzMIm)Br, was obtained in high purity as a transparent, supercooled, highly viscous liquid in multi-gram scale from *N*-methyl­imidazole and benzyl bromide in ethyl acetate solution under ambient conditions. Benzyl bromide (15.0 g, 87.7 mmol) was added in one portion to a vigorously stirred solution of *N*-methyl­imidazole (5.0 g, 60.9 mmol) in 100 ml ethyl acetate at room temperature. The clear solution became turbid after a few minutes and was stirred at room temperature overnight. Afterwards, the product was washed several times with portions of ethyl acetate and dried *in vacuo* (*T* = 90°C, *p* = 20 mbar, yield: 13.1 g, 85%).

Analytic data for (BenzMIm)Br: m.p. 72°C, EA for C_11_H_13_BrN_2_ % (calc.): C 52.47 (52.19); H 4.93 (5.18); N 10.91 (11.07); Br 31.63 (31.56).

## Refinement

Crystal data, data collection and structure refinement details are summarized in Table 1 ▸.

## Supplementary Material

Crystal structure: contains datablock(s) I. DOI: 10.1107/S2414314620007683/im4007sup1.cif

Structure factors: contains datablock(s) I. DOI: 10.1107/S2414314620007683/im4007Isup2.hkl

Supporting information file. DOI: 10.1107/S2414314620007683/im4007Isup3.cml

CCDC reference: 2009703

Additional supporting information:  crystallographic information; 3D view; checkCIF report

## Figures and Tables

**Table 1:** Experimental details

Crystal data	
Chemical formula	C_11_H_13_N_2_^+^·Br^−^
*M*_r_	253.14
Crystal system, space group	Orthorhombic, *P* *b* *c* *a*
Temperature (K)	150
*a*, *b*, *c* (Å)	10.9070 (8), 18.8993 (14), 21.6608 (15)
*V* (Å^3^)	4465.0 (6)
*Z*	16
Radiation type	Mo *K*α
μ (mm^−1^)	3.65
Crystal size (mm)	0.40 × 0.32 × 0.22

Data collection	
Diffractometer	Bruker APEXII CCD
Absorption correction	Multi-scan (*SADABS*; Bruker, 2014 ▸)
*T*_min_, *T*_max_	0.33, 0.51
No. of measured, independent and observed [*I* > 2σ(*I*)] reflections	51842, 5934, 5054
*R*_int_	0.028
(sin θ/λ)_max_ (Å^−1^)	0.682

Refinement	
*R*[*F* ^2^ > 2σ(*F* ^2^)], *wR*(*F* ^2^), *S*	0.020, 0.051, 1.03
No. of reflections	5934
No. of parameters	255
H-atom treatment	H-atom parameters constrained
Δρ_max_, Δρ_min_ (e Å^−3^)	0.32, −0.32

Computer programs: *APEX2* (Bruker, 2014 ▸), *SAINT* (Bruker, 2013 ▸), *SHELXS97* (Sheldrick, 2008 ▸), *SHELXL2018/3* (Sheldrick, 2015 ▸), *XP* in *SHELXTL* (Sheldrick, 2008 ▸) and *publCIF* (Westrip, 2010 ▸).

## full crystallographic data

### Crystal data

**Table:**

C_11_H_13_N_2_^+^·Br^−^	*D*_x_ = 1.506 Mg m^−^^3^
*M**_r_* = 253.14	Mo *K*α radiation, λ = 0.71073 Å
Orthorhombic, *P**b**c**a*	Cell parameters from 9498 reflections
*a* = 10.9070 (8) Å	θ = 2.4–30.4°
*b* = 18.8993 (14) Å	µ = 3.65 mm^−^^1^
*c* = 21.6608 (15) Å	*T* = 150 K
*V* = 4465.0 (6) Å^3^	Prism, colourless
*Z* = 16	0.40 × 0.32 × 0.22 mm
*F*(000) = 2048	

### Data collection

**Table:**

Bruker APEXII CCD diffractometer	5934 independent reflections
Radiation source: fine-focus sealed tube	5054 reflections with *I* > 2σ(*I*)
Detector resolution: 8.3333 pixels mm^-1^	*R*_int_ = 0.028
φ and ω scans	θ_max_ = 29.0°, θ_min_ = 1.9°
Absorption correction: multi-scan (SADABS; Bruker, 2014)	*h* = −14→14
*T*_min_ = 0.33, *T*_max_ = 0.51	*k* = −25→25
51842 measured reflections	*l* = −29→29

### Refinement

**Table:**

Refinement on *F*^2^	0 restraints
Least-squares matrix: full	Hydrogen site location: inferred from neighbouring sites
*R*[*F*^2^ > 2σ(*F*^2^)] = 0.020	H-atom parameters constrained
*wR*(*F*^2^) = 0.051	*w* = 1/[σ^2^(*F*_o_^2^) + (0.0243*P*)^2^ + 1.6832*P*] where *P* = (*F*_o_^2^ + 2*F*_c_^2^)/3
*S* = 1.03	(Δ/σ)_max_ = 0.002
5934 reflections	Δρ_max_ = 0.32 e Å^−^^3^
255 parameters	Δρ_min_ = −0.31 e Å^−^^3^

### Special details

**Table:**

Geometry. All esds (except the esd in the dihedral angle between two l.s. planes) are estimated using the full covariance matrix. The cell esds are taken into account individually in the estimation of esds in distances, angles and torsion angles; correlations between esds in cell parameters are only used when they are defined by crystal symmetry. An approximate (isotropic) treatment of cell esds is used for estimating esds involving l.s. planes.

### Fractional atomic coordinates and isotropic or equivalent isotropic displacement parameters (Å^2^)

**Table:**

	*x*	*y*	*z*	*U*_iso_*/*U*_eq_	
Br1	0.22716 (2)	0.01486 (2)	0.46713 (2)	0.02635 (4)	
Br2	0.78396 (2)	0.20634 (2)	0.71589 (2)	0.02478 (4)	
C1	0.17556 (12)	0.07637 (7)	0.67512 (6)	0.0210 (3)	
H1	0.234951	0.076170	0.707203	0.025*	
C2	0.10465 (12)	0.13162 (7)	0.65814 (6)	0.0205 (3)	
H2	0.104466	0.177529	0.676007	0.025*	
C3	0.05902 (12)	0.04116 (7)	0.59817 (6)	0.0204 (3)	
H3	0.022230	0.012662	0.567154	0.024*	
C4	0.19785 (14)	−0.05091 (8)	0.63992 (7)	0.0286 (3)	
H4A	0.147699	−0.080564	0.667121	0.043*	
H4B	0.281713	−0.048522	0.656022	0.043*	
H4C	0.198986	−0.071406	0.598363	0.043*	
C5	−0.05432 (12)	0.15224 (7)	0.57392 (6)	0.0228 (3)	
H5A	−0.118776	0.170949	0.601653	0.027*	
H5B	−0.094422	0.122786	0.541970	0.027*	
C6	0.01252 (12)	0.21301 (7)	0.54350 (6)	0.0213 (3)	
C7	0.00238 (14)	0.28059 (8)	0.56795 (7)	0.0271 (3)	
H7	−0.048238	0.288620	0.602946	0.033*	
C8	0.06598 (14)	0.33662 (8)	0.54144 (8)	0.0337 (3)	
H8	0.058858	0.382779	0.558435	0.040*	
C9	0.13959 (14)	0.32536 (9)	0.49039 (8)	0.0349 (4)	
H9	0.183155	0.363715	0.472431	0.042*	
C10	0.14976 (13)	0.25800 (9)	0.46543 (7)	0.0332 (3)	
H10	0.199832	0.250330	0.430171	0.040*	
C11	0.08660 (13)	0.20158 (8)	0.49200 (6)	0.0261 (3)	
H11	0.094021	0.155416	0.475052	0.031*	
C12	0.86546 (12)	0.13770 (8)	0.92679 (6)	0.0247 (3)	
H12	0.807687	0.143230	0.959254	0.030*	
C13	0.93090 (12)	0.07900 (8)	0.91414 (6)	0.0231 (3)	
H13	0.928098	0.035409	0.935940	0.028*	
C14	0.98114 (12)	0.16068 (7)	0.84610 (6)	0.0216 (3)	
H14	1.018734	0.184442	0.812405	0.026*	
C15	0.84898 (15)	0.26012 (8)	0.87971 (7)	0.0327 (3)	
H15A	0.910658	0.293794	0.894524	0.049*	
H15B	0.774922	0.264014	0.905102	0.049*	
H15C	0.828730	0.270763	0.836621	0.049*	
C16	1.08871 (12)	0.04543 (7)	0.83297 (6)	0.0220 (3)	
H16A	1.139346	0.021678	0.864745	0.026*	
H16B	1.144345	0.072702	0.805811	0.026*	
C17	1.02277 (12)	−0.01005 (7)	0.79499 (6)	0.0194 (3)	
C18	0.94292 (12)	0.00932 (7)	0.74732 (6)	0.0205 (3)	
H18	0.928300	0.057937	0.738951	0.025*	
C19	0.88501 (12)	−0.04227 (8)	0.71223 (6)	0.0253 (3)	
H19	0.831707	−0.028868	0.679570	0.030*	
C20	0.90486 (14)	−0.11361 (8)	0.72479 (7)	0.0314 (3)	
H20	0.863894	−0.148864	0.701293	0.038*	
C21	0.98439 (15)	−0.13301 (8)	0.77157 (7)	0.0320 (3)	
H21	0.998734	−0.181666	0.779895	0.038*	
C22	1.04355 (13)	−0.08133 (7)	0.80652 (7)	0.0256 (3)	
H22	1.098428	−0.094963	0.838439	0.031*	
N1	0.14580 (10)	0.02040 (6)	0.63731 (5)	0.0199 (2)	
N2	0.03241 (10)	0.10849 (6)	0.60977 (5)	0.0190 (2)	
N3	0.89775 (10)	0.18811 (6)	0.88402 (5)	0.0229 (2)	
N4	1.00287 (10)	0.09437 (6)	0.86338 (5)	0.0196 (2)	

### Atomic displacement parameters (Å^2^)

**Table:**

	*U*^11^	*U*^22^	*U*^33^	*U*^12^	*U*^13^	*U*^23^
Br1	0.02669 (8)	0.02857 (8)	0.02379 (7)	−0.00178 (6)	−0.00049 (5)	−0.00815 (5)
Br2	0.02648 (7)	0.02405 (7)	0.02380 (7)	−0.00402 (6)	0.00081 (5)	0.00084 (5)
C1	0.0192 (6)	0.0264 (7)	0.0175 (6)	−0.0047 (5)	0.0002 (5)	0.0005 (5)
C2	0.0216 (6)	0.0225 (7)	0.0175 (6)	−0.0051 (5)	0.0006 (5)	−0.0012 (5)
C3	0.0212 (6)	0.0213 (7)	0.0186 (6)	−0.0025 (5)	0.0009 (5)	−0.0004 (5)
C4	0.0297 (7)	0.0229 (7)	0.0331 (7)	0.0038 (6)	−0.0004 (6)	0.0018 (6)
C5	0.0206 (6)	0.0235 (7)	0.0243 (6)	0.0001 (5)	−0.0023 (5)	0.0022 (5)
C6	0.0197 (6)	0.0223 (7)	0.0221 (6)	0.0010 (5)	−0.0044 (5)	0.0034 (5)
C7	0.0272 (7)	0.0255 (7)	0.0286 (7)	0.0018 (6)	−0.0040 (6)	−0.0005 (6)
C8	0.0321 (8)	0.0213 (7)	0.0477 (9)	−0.0010 (6)	−0.0119 (7)	0.0043 (7)
C9	0.0231 (7)	0.0342 (9)	0.0473 (9)	−0.0041 (6)	−0.0090 (6)	0.0203 (7)
C10	0.0211 (7)	0.0478 (10)	0.0307 (7)	0.0035 (6)	0.0009 (6)	0.0150 (7)
C11	0.0237 (7)	0.0298 (8)	0.0246 (6)	0.0051 (6)	−0.0013 (5)	0.0022 (6)
C12	0.0204 (6)	0.0349 (8)	0.0190 (6)	−0.0056 (6)	0.0010 (5)	−0.0042 (5)
C13	0.0241 (7)	0.0272 (7)	0.0179 (6)	−0.0067 (6)	0.0010 (5)	0.0009 (5)
C14	0.0229 (6)	0.0222 (7)	0.0197 (6)	−0.0023 (5)	0.0010 (5)	−0.0004 (5)
C15	0.0351 (8)	0.0265 (8)	0.0365 (8)	0.0071 (7)	−0.0044 (6)	−0.0079 (6)
C16	0.0194 (6)	0.0239 (7)	0.0227 (6)	0.0021 (5)	0.0003 (5)	−0.0008 (5)
C17	0.0176 (6)	0.0203 (6)	0.0203 (6)	0.0003 (5)	0.0042 (5)	0.0007 (5)
C18	0.0184 (6)	0.0212 (6)	0.0218 (6)	0.0016 (5)	0.0036 (5)	0.0026 (5)
C19	0.0198 (6)	0.0322 (8)	0.0240 (6)	−0.0006 (6)	0.0008 (5)	−0.0007 (6)
C20	0.0319 (8)	0.0272 (8)	0.0350 (8)	−0.0043 (6)	0.0039 (6)	−0.0095 (6)
C21	0.0377 (8)	0.0179 (7)	0.0404 (8)	0.0030 (6)	0.0052 (7)	0.0011 (6)
C22	0.0267 (7)	0.0230 (7)	0.0273 (6)	0.0038 (6)	0.0013 (6)	0.0056 (5)
N1	0.0195 (5)	0.0213 (6)	0.0189 (5)	−0.0012 (4)	0.0014 (4)	0.0008 (4)
N2	0.0191 (5)	0.0202 (6)	0.0176 (5)	−0.0021 (4)	−0.0005 (4)	0.0004 (4)
N3	0.0212 (5)	0.0249 (6)	0.0226 (5)	−0.0005 (5)	−0.0004 (4)	−0.0050 (5)
N4	0.0195 (5)	0.0216 (6)	0.0178 (5)	−0.0015 (4)	0.0004 (4)	−0.0007 (4)

### Geometric parameters (Å, º)

**Table:**

C1—C2	1.3504 (19)	C12—C13	1.347 (2)
C1—N1	1.3767 (17)	C12—N3	1.3747 (18)
C1—H1	0.9500	C12—H12	0.9500
C2—N2	1.3820 (16)	C13—N4	1.3819 (16)
C2—H2	0.9500	C13—H13	0.9500
C3—N2	1.3291 (17)	C14—N4	1.3293 (17)
C3—N1	1.3298 (16)	C14—N3	1.3307 (17)
C3—H3	0.9500	C14—H14	0.9500
C4—N1	1.4636 (18)	C15—N3	1.4643 (19)
C4—H4A	0.9800	C15—H15A	0.9800
C4—H4B	0.9800	C15—H15B	0.9800
C4—H4C	0.9800	C15—H15C	0.9800
C5—N2	1.4771 (17)	C16—N4	1.4717 (17)
C5—C6	1.5115 (19)	C16—C17	1.5144 (18)
C5—H5A	0.9900	C16—H16A	0.9900
C5—H5B	0.9900	C16—H16B	0.9900
C6—C7	1.3871 (19)	C17—C22	1.3887 (19)
C6—C11	1.3943 (19)	C17—C18	1.3995 (18)
C7—C8	1.390 (2)	C18—C19	1.388 (2)
C7—H7	0.9500	C18—H18	0.9500
C8—C9	1.383 (2)	C19—C20	1.392 (2)
C8—H8	0.9500	C19—H19	0.9500
C9—C10	1.388 (2)	C20—C21	1.383 (2)
C9—H9	0.9500	C20—H20	0.9500
C10—C11	1.394 (2)	C21—C22	1.394 (2)
C10—H10	0.9500	C21—H21	0.9500
C11—H11	0.9500	C22—H22	0.9500
			
C2—C1—N1	107.28 (11)	N4—C14—N3	108.38 (12)
C2—C1—H1	126.4	N4—C14—H14	125.8
N1—C1—H1	126.4	N3—C14—H14	125.8
C1—C2—N2	106.76 (12)	N3—C15—H15A	109.5
C1—C2—H2	126.6	N3—C15—H15B	109.5
N2—C2—H2	126.6	H15A—C15—H15B	109.5
N2—C3—N1	108.50 (11)	N3—C15—H15C	109.5
N2—C3—H3	125.7	H15A—C15—H15C	109.5
N1—C3—H3	125.7	H15B—C15—H15C	109.5
N1—C4—H4A	109.5	N4—C16—C17	112.10 (10)
N1—C4—H4B	109.5	N4—C16—H16A	109.2
H4A—C4—H4B	109.5	C17—C16—H16A	109.2
N1—C4—H4C	109.5	N4—C16—H16B	109.2
H4A—C4—H4C	109.5	C17—C16—H16B	109.2
H4B—C4—H4C	109.5	H16A—C16—H16B	107.9
N2—C5—C6	110.22 (11)	C22—C17—C18	119.21 (13)
N2—C5—H5A	109.6	C22—C17—C16	119.76 (12)
C6—C5—H5A	109.6	C18—C17—C16	121.01 (12)
N2—C5—H5B	109.6	C19—C18—C17	120.22 (13)
C6—C5—H5B	109.6	C19—C18—H18	119.9
H5A—C5—H5B	108.1	C17—C18—H18	119.9
C7—C6—C11	119.62 (13)	C18—C19—C20	120.15 (13)
C7—C6—C5	119.66 (12)	C18—C19—H19	119.9
C11—C6—C5	120.70 (13)	C20—C19—H19	119.9
C6—C7—C8	120.26 (14)	C21—C20—C19	119.82 (14)
C6—C7—H7	119.9	C21—C20—H20	120.1
C8—C7—H7	119.9	C19—C20—H20	120.1
C9—C8—C7	120.18 (15)	C20—C21—C22	120.15 (14)
C9—C8—H8	119.9	C20—C21—H21	119.9
C7—C8—H8	119.9	C22—C21—H21	119.9
C8—C9—C10	119.95 (14)	C17—C22—C21	120.42 (13)
C8—C9—H9	120.0	C17—C22—H22	119.8
C10—C9—H9	120.0	C21—C22—H22	119.8
C9—C10—C11	120.09 (14)	C3—N1—C1	108.69 (11)
C9—C10—H10	120.0	C3—N1—C4	124.92 (12)
C11—C10—H10	120.0	C1—N1—C4	126.38 (11)
C10—C11—C6	119.89 (14)	C3—N2—C2	108.76 (11)
C10—C11—H11	120.1	C3—N2—C5	125.20 (11)
C6—C11—H11	120.1	C2—N2—C5	125.92 (11)
C13—C12—N3	107.33 (12)	C14—N3—C12	108.73 (12)
C13—C12—H12	126.3	C14—N3—C15	124.82 (12)
N3—C12—H12	126.3	C12—N3—C15	126.45 (12)
C12—C13—N4	106.84 (12)	C14—N4—C13	108.72 (11)
C12—C13—H13	126.6	C14—N4—C16	125.44 (11)
N4—C13—H13	126.6	C13—N4—C16	125.83 (12)
			
N1—C1—C2—N2	−0.17 (14)	C20—C21—C22—C17	0.4 (2)
N2—C5—C6—C7	−103.83 (14)	N2—C3—N1—C1	−0.01 (14)
N2—C5—C6—C11	74.65 (15)	N2—C3—N1—C4	178.70 (12)
C11—C6—C7—C8	−0.2 (2)	C2—C1—N1—C3	0.12 (14)
C5—C6—C7—C8	178.27 (13)	C2—C1—N1—C4	−178.57 (12)
C6—C7—C8—C9	0.2 (2)	N1—C3—N2—C2	−0.10 (14)
C7—C8—C9—C10	0.2 (2)	N1—C3—N2—C5	176.21 (11)
C8—C9—C10—C11	−0.5 (2)	C1—C2—N2—C3	0.17 (14)
C9—C10—C11—C6	0.4 (2)	C1—C2—N2—C5	−176.11 (12)
C7—C6—C11—C10	−0.1 (2)	C6—C5—N2—C3	−116.36 (14)
C5—C6—C11—C10	−178.54 (12)	C6—C5—N2—C2	59.32 (16)
N3—C12—C13—N4	−0.04 (15)	N4—C14—N3—C12	0.03 (15)
N4—C16—C17—C22	−123.69 (13)	N4—C14—N3—C15	−179.51 (12)
N4—C16—C17—C18	57.74 (16)	C13—C12—N3—C14	0.01 (15)
C22—C17—C18—C19	0.23 (19)	C13—C12—N3—C15	179.54 (13)
C16—C17—C18—C19	178.81 (12)	N3—C14—N4—C13	−0.05 (14)
C17—C18—C19—C20	0.8 (2)	N3—C14—N4—C16	179.45 (11)
C18—C19—C20—C21	−1.3 (2)	C12—C13—N4—C14	0.06 (15)
C19—C20—C21—C22	0.7 (2)	C12—C13—N4—C16	−179.44 (12)
C18—C17—C22—C21	−0.8 (2)	C17—C16—N4—C14	−105.12 (14)
C16—C17—C22—C21	−179.41 (13)	C17—C16—N4—C13	74.30 (15)
